# Pulmonary Mucormycosis in Diabetic Patients: A Case Series From a Tertiary Respiratory Center in Sri Lanka

**DOI:** 10.7759/cureus.88260

**Published:** 2025-07-18

**Authors:** Kasun T Maduranga, Arthihai Srirangan, P.K.E. Diyalagoda, Sumudu Palihawadana, Eshantha Perara

**Affiliations:** 1 Department of Pulmonary Medicine, National Hospital for Respiratory Diseases, Welisara, LKA; 2 Department of Radiology, National Hospital for Respiratory Diseases, Welisara, LKA

**Keywords:** histopathologic diagnosis, liposomal amphotericin b, pulmonary fungal infections, pulmonary mucormycosis, type 2 diabetes mellitus

## Abstract

Pulmonary mucormycosis is a rare but aggressive fungal infection that primarily affects immunocompromised individuals, especially those with uncontrolled diabetes mellitus. Its non-specific presentation can mimic more common pulmonary conditions, often resulting in delayed diagnosis and treatment. Here, we present a case series of three Sri Lankan patients with type 2 diabetes mellitus who developed pulmonary mucormycosis. Each case demonstrated variable respiratory and constitutional symptoms. Imaging, primarily chest radiography and contrast-enhanced CT, revealed findings such as cavitary lesions and pleural effusions. Diagnosis was confirmed via bronchoscopic or surgical sampling, with fungal stains showing broad, non-septate hyphae characteristic of mucormycosis. Two patients were successfully treated with prolonged intravenous liposomal amphotericin B therapy. One patient, however, rapidly deteriorated and died despite early antifungal initiation. This series underscores the importance of early clinical suspicion and tissue diagnosis in suspected invasive fungal infections among diabetic patients. In resource-limited and endemic settings, timely recognition and intervention remain crucial for improving patient outcomes.

## Introduction

Pulmonary mucormycosis is a rare but aggressive, opportunistic fungal infection caused by organisms of the order Mucorales [1]. It carries a high morbidity and mortality, particularly among individuals with uncontrolled diabetes mellitus, hematologic malignancies, or those receiving immunosuppressive therapy [2]. Due to its non-specific clinical and radiological features, it is frequently misdiagnosed as more common pulmonary conditions, such as tuberculosis or bacterial pneumonia, leading to potentially dangerous delays in diagnosis and treatment [2].

In recent years, the incidence of pulmonary mucormycosis has been increasingly recognized in diabetic populations, particularly in tropical and resource-limited settings [2,3]. Clinical manifestations are often subtle or non-specific and may include fever, cough, hemoptysis, and weight loss. While some patients may present atypically, others exhibit classical signs suggestive of mucormycosis, highlighting the variability in clinical presentation. Imaging studies typically reveal cavitary lesions, consolidation, or pleural involvement; however, definitive diagnosis requires tissue sampling with histopathological or microbiological confirmation [4].

Early diagnosis, prompt antifungal therapy typically with liposomal amphotericin B, and surgical debridement, when feasible, are essential for improving survival [5]. Nevertheless, the prognosis remains poor, especially in cases with delayed presentation or disseminated disease.

In this case series, we present three patients with confirmed pulmonary mucormycosis treated at a tertiary respiratory center in Sri Lanka. These cases illustrate the diverse clinical spectrum, diagnostic challenges, and outcomes associated with this life-threatening infection, underscoring the importance of early clinical suspicion in high-risk populations.

## Case presentation

Case 1

A 66-year-old Sri Lankan woman presented with a one-week history of worsening cough and exertional dyspnea. She also reported low-grade fever, anorexia, and unintentional weight loss of 4 kg over the preceding two months. Her medical history included type 2 diabetes mellitus, hypertension, and ischemic heart disease. Notably, she had experienced cavitary pneumonia five months earlier, which was managed with empirical antibiotics.

On examination, she was tachycardic with a heart rate of 120 beats per minute and a blood pressure of 120/75 mmHg. Her body mass index was 21 kg/m^2^. Respiratory examination revealed absent breath sounds and stony dullness to percussion over the right middle and lower lung zones. Cardiovascular, abdominal, and neurological examinations were unremarkable.

**Figure 1:**
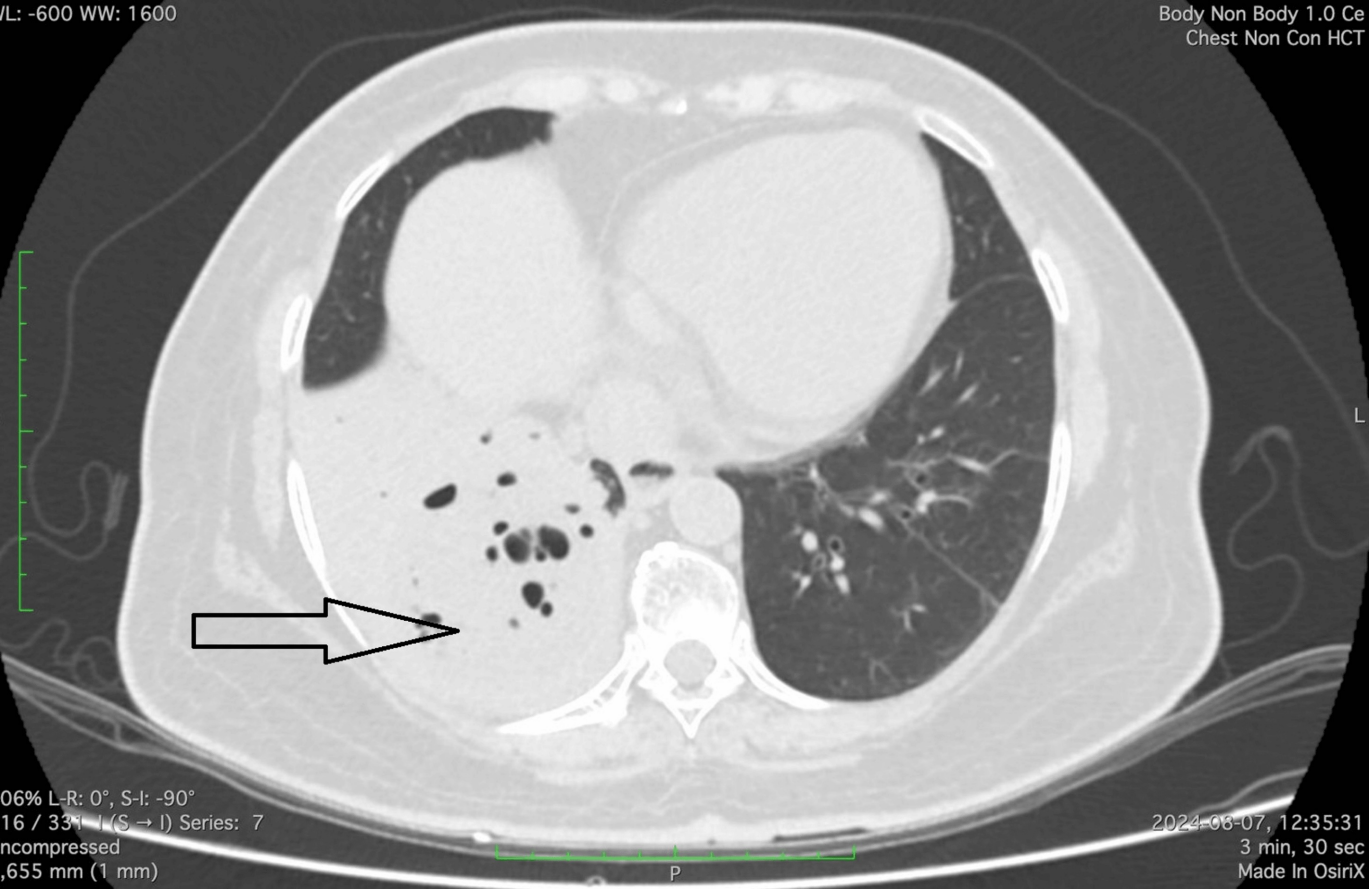
High-resolution CT of the chest revealing right lower lobe consolidation with central necrosis (black arrow), consistent with necrotizing pneumonia.

Chest radiography and ultrasonography showed a moderate right-sided pleural effusion. Pleural fluid analysis revealed an exudative effusion with neutrophilic predominance, low adenosine deaminase level, and no malignant cells, suggestive of an infective etiology. High-resolution CT of the chest demonstrated right lower lobe consolidation with central necrosis (Figure 1). Due to persistent symptoms and radiological findings, the patient underwent video-assisted thoracoscopic surgery for decortication. Intraoperatively, a large lung abscess and a bronchopulmonary fistula were identified. Histopathology showed broad, ribbon-like, non-septate fungal hyphae consistent with mucormycosis, and fungal culture confirmed the diagnosis.

She was started on intravenous liposomal amphotericin B at a dose of 3 mg/kg/day, which was continued for 56 days. During treatment, she developed several episodes of hypokalemia and hypomagnesemia, which were managed successfully. She showed gradual clinical and radiological improvement and remains under regular respiratory follow-up, with no signs of recurrence. Relevant laboratory values are summarized in Table 1.

**Table 1:** Summary of blood and sputum investigations of the three cases. ADA = adenosine deaminase; c-ANCA = cytoplasmic antineutrophil cytoplasmic antibodies; p-ANCA = perinuclear antineutrophil cytoplasmic antibodies; AFB = acid-fast bacilli

Investigation	Reference range	Case 1	Case 2	Case 3
White blood cells (×10⁹/L)	4.0–11.0	29.9	6.95	18.0
Neutrophils (%)	40–75	95	80	70
Hemoglobin (g/dL)	12.0–16.0 (female)	7.8	10.5	8.7
Platelets (×10⁹/L)	150–450	405	122	284
C-reactive protein (mg/L)	<5	225	39	180
Erythrocyte sedimentation rate (mm/hour)	<20 (female)	68	37	83
Lactate dehydrogenase (U/L)	140–280	13,719	—	—
Pleural fluid cell count	—	3,200 (90% neutrophils)	—	—
Pleural fluid ADA (U/L)	<40	10	—	—
c-ANCA/p-ANCA	Negative	—	—	Negative
Sputum AFB	Negative	Negative	Negative	Negative

Case 2

A 61-year-old female with a history of type 2 diabetes mellitus and hypertension presented with a two-week history of hemoptysis, low-grade fever, anorexia, and weight loss. On admission, her vital signs were stable (blood pressure, 140/90 mmHg; pulse, 78 beats per minute; respiratory rate, 24 breaths per minute; SpO₂, 100% on room air). Lung auscultation revealed scattered crepitation. Abdominal and neurological examinations were unremarkable.

**Figure 2:**
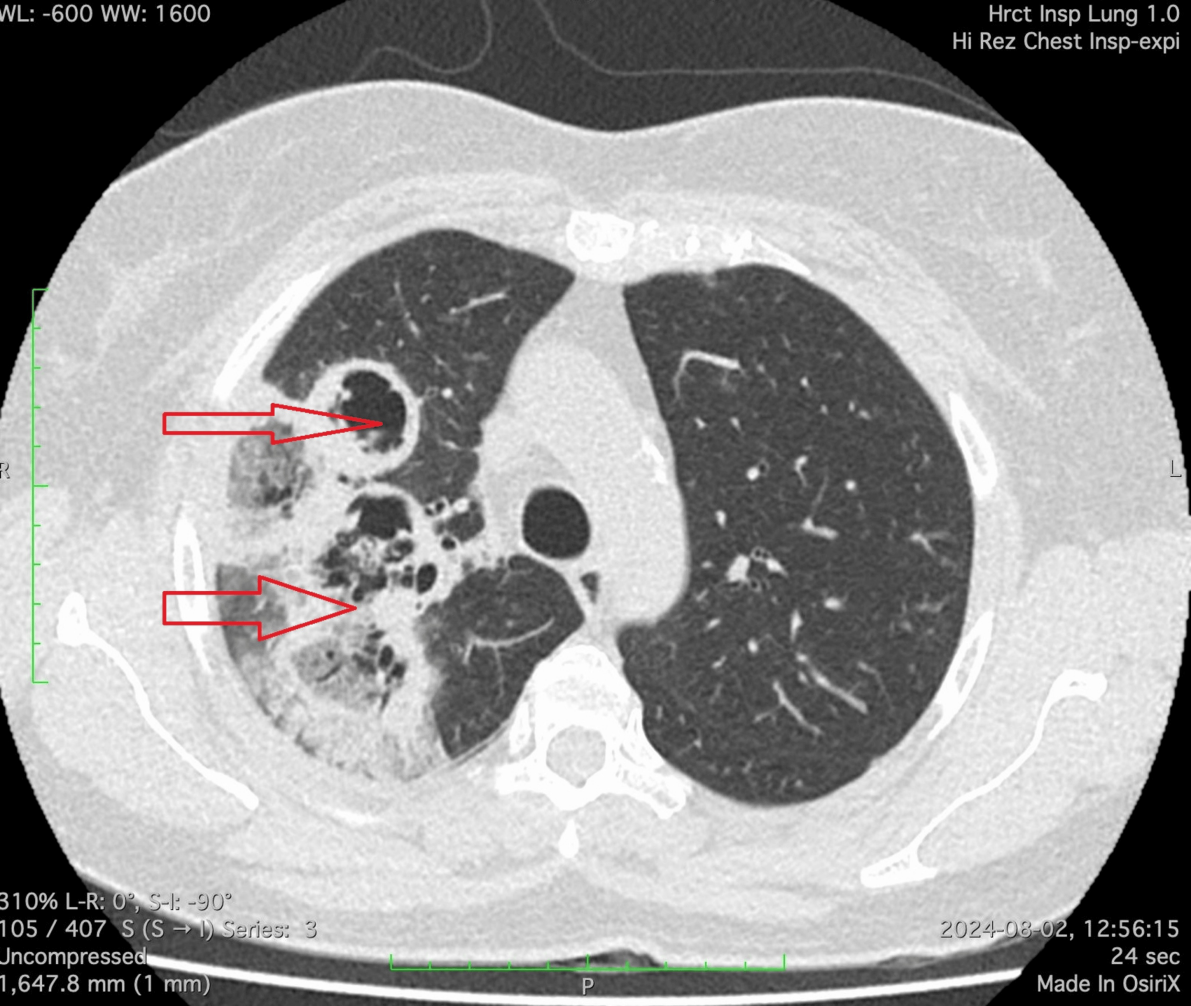
Contrast-enhanced CT showing a thick-walled cavitary lesion in the right upper lobe (red arrow). The internal content appears heterogeneous, with necrotic areas. Surrounding consolidation and mild ground glass opacities can be seen, suggestive of an aggressive infectious process.

Chest radiography revealed cavitary lesions in the right lung. Further evaluation with contrast-enhanced CT (CECT) demonstrated a thick-walled cavitary lesion in the right upper lobe (Figure 2). Flexible bronchoscopy was performed, and bronchoalveolar lavage (BAL) confirmed the presence of broad, non-septate fungal hyphae, consistent with pulmonary mucormycosis.

She was treated with intravenous liposomal amphotericin B (3 mg/kg/day) for 56 days. Multiple episodes of hypokalemia and hypomagnesemia occurred but were successfully managed. She made a full clinical recovery and remains well on regular follow-up with no recurrence. Relevant laboratory values are summarized in Table 1.

Case 3

A 55-year-old male with poorly controlled type 2 diabetes mellitus presented with a four-week history of persistent fever, productive cough, and constitutional symptoms, including weight and appetite loss. He subsequently developed hemoptysis.

On admission, his vital signs indicated hemodynamic compromise, with a blood pressure of 90/60 mmHg, a pulse rate of 110 beats per minute, and an oxygen saturation of 94% on 4 L/minute supplemental oxygen. Lung auscultation revealed bilateral crepitation. Neurological and abdominal examinations were unremarkable.

**Figure 3:**
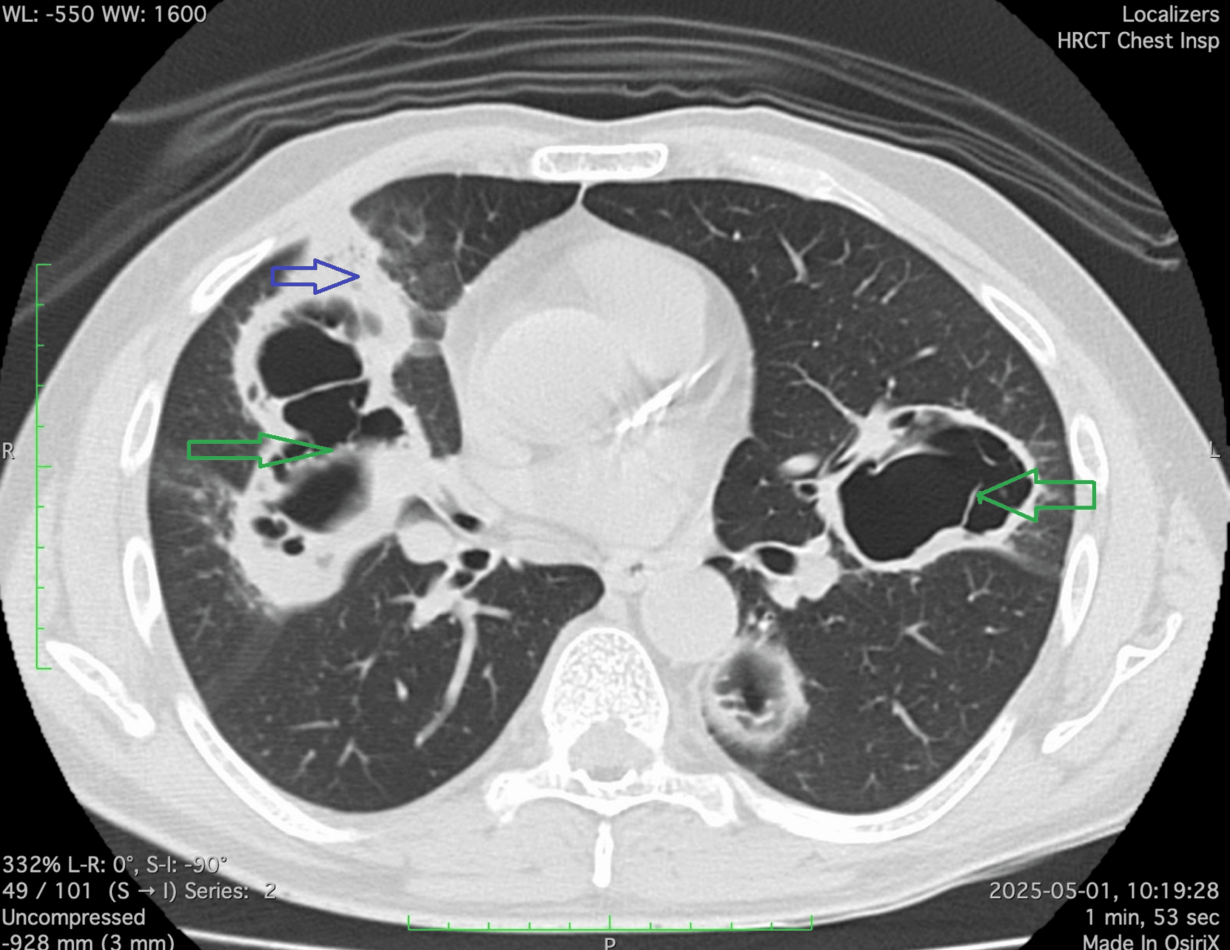
Contrast-enhanced CT of the chest demonstrating bilateral cavitary lesions (green arrow), predominantly in the upper lobes, with surrounding ground glass-opacities (blue arrow). Cavity walls are thick and irregular, with some showing air-fluid levels suggestive of secondary infection. The findings are consistent with invasive fungal infection with widespread parenchymal involvement.

Chest radiography revealed bilateral cavitary lesions. Serologic testing for vasculitis, including cytoplasmic antineutrophil cytoplasmic antibodies and perinuclear antineutrophil cytoplasmic antibodies, was negative. The CECT of the chest demonstrated bilateral cavitary lesions (Figure 3) with surrounding ground-glass opacities. BAL obtained via bronchoscopy confirmed mucormycosis on fungal staining.

The patient was commenced on intravenous liposomal amphotericin B (3 mg/kg/day), but he developed acute kidney injury and significant electrolyte imbalances, including hypokalemia and hypomagnesemia. Despite close monitoring and supportive care, his condition deteriorated into shock, requiring resuscitation. Unfortunately, he succumbed to the invasive pulmonary mucormycosis and passed away one week after the initiation of antifungal therapy. Relevant laboratory values are summarized in Table 1.

## Discussion

This case series highlights three diverse presentations of pulmonary mucormycosis in diabetic patients, underscoring the diagnostic challenges and therapeutic complexities associated with this rare but aggressive fungal infection. While diabetes mellitus is a well-established predisposing factor, our findings emphasize that mucormycosis can occur even in the absence of other classical immunocompromised status, such as hematological malignancy or neutropenia.

All three patients presented with constitutional symptoms, including fever, cough, and weight loss. Hemoptysis, a hallmark of invasive fungal infection, was prominent only in the second and third cases. Interestingly, the first patient presented with a necrotizing pneumonia and pleural effusion, an uncommon manifestation of mucormycosis. Surgical exploration revealed a large lung abscess with bronchopulmonary fistula, and histopathology confirmed mucormycosis. This case illustrates the pathogen’s capacity to mimic post-infectious empyema or cavitary bacterial pneumonia, potentially delaying diagnosis.

In contrast, the second patient had a solitary thick-walled upper lobe cavity, radiologically indistinguishable from tuberculosis or post-COVID-19 fungal infection. Early bronchoscopy with BAL enables a non-invasive diagnosis, allowing initiation of antifungal therapy without the need for surgery. This highlights the critical role of early bronchoscopy evaluation and fungal staining in high-risk patients, particularly when imaging findings are inconclusive.

The third case represents fulminant pulmonary mucormycosis with bilateral cavitary lesions and systemic involvement, progressing to hypoxic respiratory failure and shock. Despite the early initiation of liposomal amphotericin B, the patient developed acute kidney injury and succumbed to refractory shock. This fatal outcome underscores the aggregate nature of the limited therapeutic window, even with prompt antifungal therapy.

A central theme across these cases is the diagnostic delay often encountered in pulmonary mucormycosis. All three patients had negative workups for tuberculosis, and mucormycosis was only considered after persistent symptoms, atypical imaging, and invasive diagnostic procedures. Notably, none of the patients were neutropenic or receiving corticosteroids, reinforcing that pulmonary mucormycosis should be considered in diabetic patients with atypical pneumonia or cavitatory lung diseases, regardless of the immunosuppression status.

Liposomal amphotericin B was used in all cases, with a duration ranging from one to eight weeks. Electrolyte disturbances, especially hypokalemia and hypomagnesemia, were universally observed during treatment, requiring close monitoring and correction.

In our case series, outcomes varied. One patient achieved full recovery with combined surgery and medical therapy, another recovered with medical therapy alone, and one died despite early treatment. These outcomes reflect the unpredictable clinical course of mucormycosis and its high morbidity and mortality, even with appropriate management.

To our knowledge, this is the first Sri Lankan case series describing pulmonary mucormycosis in diabetic individuals without a history of organ or stem cell transplantation and underlying malignancy. It demonstrates the spectrum of clinical presentations, from localized cavitary disease to fulminant bilateral involvement. Importantly, this series highlights the value of early bronchoscopy in establishing a diagnosis and the potential to avoid surgery in selected cases. Furthermore, it underscores the clinical need for heightened clinical suspicion and timely intervention to improve outcomes in this highly lethal infection.

## Conclusions

Pulmonary mucormycosis, though uncommon, should be actively considered in diabetic patients presenting with persistent or atypical respiratory symptoms, particularly when imaging reveals cavitary lesions or necrotizing pneumonia. Early diagnosis via bronchoscopy or surgical sampling, combined with timely initiation of antifungal therapy, can significantly influence outcomes. This case series highlights the varied clinical spectrum of this disease, the utility of BAL in diagnosis, and the critical role of early intervention. In resource-limited settings such as Sri Lanka, timely recognition and early diagnostic workup are crucial to minimizing the morbidity and mortality associated with this potentially fatal infection.
